# P-100. Durable immune response induced by self-amplifying mRNA (samRNA) SARS-CoV-2 vaccine candidates against variants of concern (VOC) in HIV-negative and people living with HIV (PLWH) populations in South Africa, irrespective of prior SARS-CoV-2 vaccination

**DOI:** 10.1093/ofid/ofae631.307

**Published:** 2025-01-29

**Authors:** Atul Nagare, Elizabeth Martin, A Koen, E Mitha, Khatija Ahmed, M Dhar, M Marrali, Meghan Hart, Harshni Venkatraman, Jason Jaroslavsky, Jenchun Kuan, Sonia Kounlavouth, Enrique Podaza, Mathieu Le Gars, A Allen, Karin Jooss, Sabhir Madhi

**Affiliations:** Gritstone bio, Inc, San Jose, California; Gritstone bio, Inc., Manasquan, New Jersey; Wits Vaccines & Infectious Diseases Analytics (VIDA) Research Unit, South Africa – Johannesburg (South Africa), Johannesburg, Gauteng, South Africa; Newtown Clinical Research Centre, South Africa - Newtown (South Africa), Newtown, Gauteng, South Africa; Setshaba Research Centre, Praetoria, Gauteng, South Africa; Shandukani Research - Johannesburg (South Africa), Johannesburg, Gauteng, South Africa; Gritstone bio, Inc, San Jose, California; Gritstone bio, Inc, San Jose, California; Gritstone Bio, Cambridge, Massachusetts; Gritstone Bio, Cambridge, Massachusetts; Gritstone bio, Inc, San Jose, California; Gritstone Bio, Cambridge, Massachusetts; Gritstone bio, Inc, San Jose, California; Gritstone bio, Inc, San Jose, California; Gritstone bio, Inc, San Jose, California; Gritstone Bio, Cambridge, Massachusetts; Wits Vaccines & Infectious Diseases Analytics (VIDA) Research Unit, Johannesburg, Gauteng, South Africa

## Abstract

**Background:**

Protection against COVID-19 from authorized SARS-CoV-2 vaccines diminishes after six months, so it is evident we need a second-generation vaccine that provides durable cross-variant protection through durable antibody responses and/or T cell responses to conserved epitopes. CORAL-CEPI (NCT05435027) is a Phase I study conducted in South Africa evaluating three self-amplifying mRNA (samRNA)-based SARS-CoV-2 vaccine candidates.

**Figure 1:**
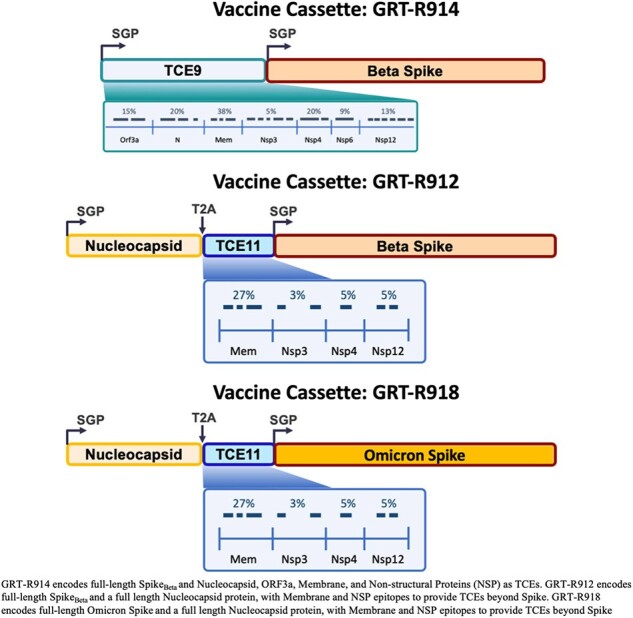
Vaccine Cassette Design

GRT-R914 encodes full length SpikeBeta and T cell epitopes (TCE) from conserved viral proteins (TCE9). GRT-R912 encodes full length SpikeBeta, full length Nucleocapsid, and a TCE cassette (TCE11). GRT-R918 encodes full length SpikeOmicron BA.1, full length Nucleocapsid, and a TCE cassette (TCE11). SGP: Sub-genomic promoter; T2A: Ribosomal skip-site

**Methods:**

Vaccine candidates GRT-R914, GRT-R912, and GRT-R918 encode full-length Spike (Beta or Omicron BA.1), Nucleocapsid (full-length or selected T cell epitopes [TCEs]), and non-Spike TCE’s from conserved viral proteins beyond Spike (Fig.1). GRT-R914 and GRT-R912 were evaluated in HIV-negative and PLWH populations who were SARS-CoV-2 anti-Spike and anti-Nucleocapsid seronegative or seropositive at baseline (Parts A/B/C). In Part D, GRT-R918 was evaluated in adults who were either previously vaccinated against SARS-CoV-2 or vaccine naïve (Fig.2). Primary objectives included safety assessments (reactogenicity and adverse events [AEs]). Secondary objectives assessed ancestral Spike-specific binding IgG (bAb) and neutralizing antibodies (nAbs) to SARS-CoV-2 variants as well as T cell responses against Spike and TCEs.

**Figure 2:**
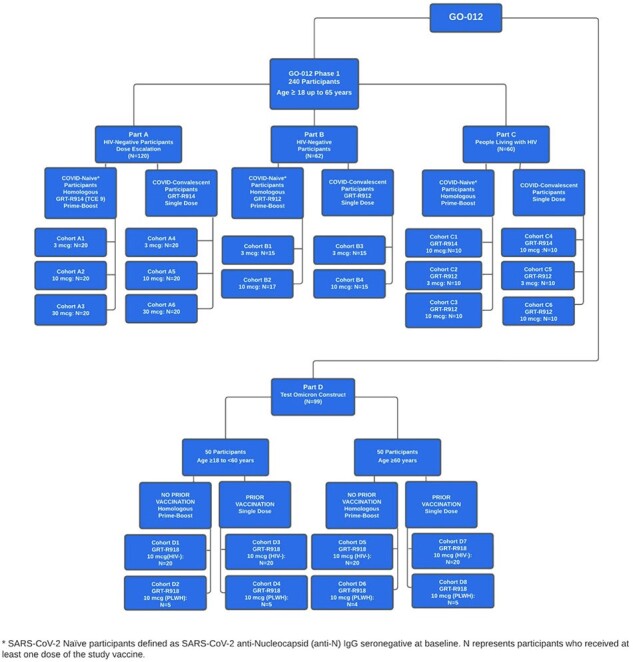
Study Design GO-012 Study Schema (Parts A, B, C, and D): 341 participants received either GRT-R914, GRT-R912, or GRT-R918 vaccine candidates. 140 participants received GRT-R914 (3mcg, 10mcg, or 30mcg), 102 participants received GRT-R912 (3mcg or 10mcg), and 99 participants received GRT-R918 (10mcg)

**Results:**

Among 341 vaccinated participants, most reported reactogenicity events were grade 1 or 2 and transient in nature. No vaccine-related serious AEs or severe COVID-19 cases were reported. Twenty-five out of 341 participants reported grade 3 solicited AE’s which resolved within 1-4 days. IgG titers against WT and nAb titers against Beta, Delta, and Omicron BA.1 were increased after administration of any of the three vaccine candidates and were durable up to at least 12 months in both HIV negative individuals and PLWH (Fig. 3 and 4). Spike and/or TCE-specific T cell responses were increased or maintained in the majority of participants after vaccination.

**Figure 3:**
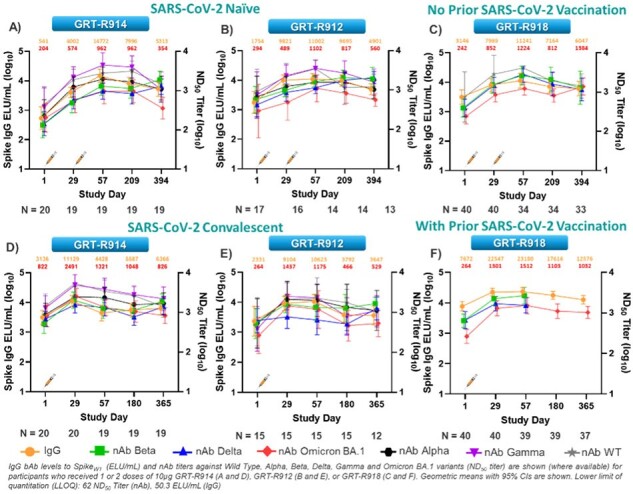
Results SpikeWT IgG bAbs and nAb titers against Alpha, Beta, Delta, Gamma, and Omicron BA.1 variants are induced and maintained through at least 12-months in HIV-negative participants irrespective of SARS-CoV-2 serostatus or vaccination status at baseline

**Conclusion:**

All dose levels of all samRNA vaccine candidates were generally well-tolerated. Administration of any of the three vaccine candidates induced robust and durable IgG binding antibodies to Wild Type (WT) and nAbs against variants of concern and T cell responses to both Spike and TCE epitopes. Complete study results will be presented.

**Figure 4:**
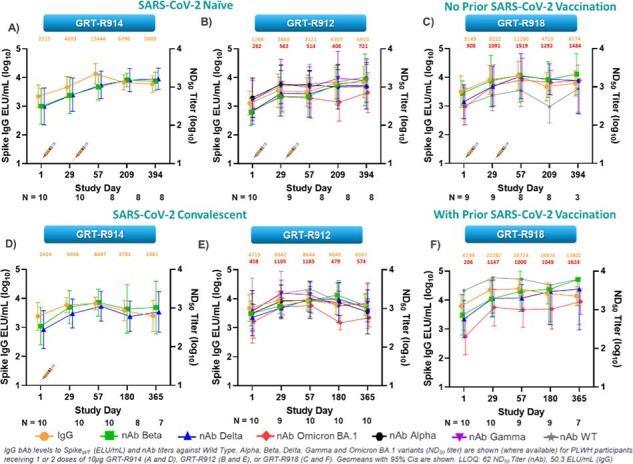
Results IgG levels to SpikeWT and nAb titers to Alpha, Beta, Delta, Gamma, and Omicron BA.1 variants are increased and remain durable through 12 months in PLWH following 10µg dose(s) of GRT-R914, GRT-R912, or GRT-R918

**Disclosures:**

**Atul Nagare, MBBS**, Gritstone bio: employee **Elizabeth Martin, DO, MPH, MBA**, Gritstone bio: employee **M. Marrali, PhD**, Gritstone bio: employee **Meghan Hart, MS**, Gritstone bio: employee **Harshni Venkatraman, MS**, Gritstone bio: employee **Jason Jaroslavsky, MS**, Gritstone bio: employee **Jenchun Kuan, PhD**, Gritstone bio: employee **Sonia Kounlavouth, BS**, Gritstone bio: employee **Enrique Podaza, PhD**, Gritstone bio: employee **Mathieu Le Gars, PhD**, Gritstone bio: employee **A. Allen, MBBS, PhD**, Gritstone bio: Board Member|Gritstone bio: Stocks/Bonds (Public Company) **Karin Jooss, PhD**, Gritstone bio: employee|Gritstone bio: Stocks/Bonds (Public Company)

